# Targeting USP-7 by a Novel Fluorinated 5-Pyrazolyl-Urea Derivative

**DOI:** 10.3390/ijms24119200

**Published:** 2023-05-24

**Authors:** Elva Morretta, Chiara Brullo, Raffaella Belvedere, Antonello Petrella, Andrea Spallarossa, Maria Chiara Monti

**Affiliations:** 1 Department of Pharmacy, University of Salerno, Via Giovanni Paolo II, Fisciano, 84084 Salerno, Italy; emorretta@unisa.it (E.M.); rbelvedere@unisa.it (R.B.); apetrella@unisa.it (A.P.); 2 Department of Pharmacy, University of Genova, Viale Benedetto XV, 3, 16132 Genova, Italy; chiara.brullo@unige.it (C.B.); andrea.spallarossa@unige.it (A.S.)

**Keywords:** pyrazole derivative, DARTS, t-LIP-MRM, USP-7, neuroblastoma, molecular docking

## Abstract

The impact of innovative technologies on the target discovery has been employed here to characterize the interactome of STIRUR 41, a promising 3-fluoro-phenyl-5-pyrazolyl-urea derivative endowed with anti-cancer activity, on neuroblastoma-related cells. A drug affinity responsive target stability-based proteomic platform has been optimized to elucidate the molecular mechanism at the basis of STIRUR 41 action, together with immunoblotting analysis and in silico molecular docking. Ubiquitin Specific Protease 7 (USP-7), one of the deubiquitinating enzymes which protect substrate proteins from proteasomal degradation, has been identified as the most affine STIRUR 41 target. As further demonstrated by in vitro and in-cell assays, STIRUR 41 was able to inhibit both the enzymatic activity of USP-7 and its expression levels in neuroblastoma-related cells, thus laying an encouraging base for the blockade of USP-7 downstream signaling.

## 1. Introduction

Neuroblastoma (NB) is an aggressive pediatric tumor; it is the most common cancer in babies and the third most common cancer in children, after leukemia and brain tumor. About 90% of cases occur in children less than 5 years old, and about 15% of cancer deaths in children are due to NB. The cancer is divided into low-, intermediate-, and high-risk groups grounded on age and cancer stage [1]. Treatment and outcomes depend on the risk group, and they embrace surgery, radiation, chemotherapy, or stem cell transplantation. In high-risk diseases, probabilities of survival are low [2].

At a biochemical level, NB is characterized by an elevated flow of chemokines promoting neutrophils which shift to the tumor site [3]. In 25% of NB cases, there is a genetic mutation which is the amplification of the *MYCN* gene [4], the main genetic marker prognostic of the most aggressive forms, with a high ability to metastasize and develop resistance to conventional therapies [5]. It has also been revealed that HTLA-230 cells, which are stage-IV NB cells with *MYCN* amplification, own a good plasticity [6], and they are associated with a high tumor vascularity [7]. Ubiquitin-specific protease 7 (USP-7; also known as HAUSP) has been demonstrated asana *MYCN* function regulator in neuroblastoma [8].

**Figure 1:**
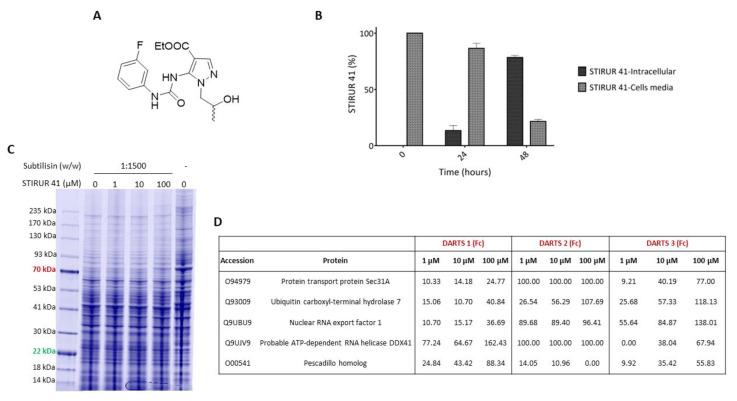
(**A**) STIRUR 41 chemical structure. (**B**) STIRUR 41 cell permeability was evaluated, quantifying the internalized molecule (dark gray bars) and the residual one in the cell’s media (light gray bars). (**C**) Coomassie-stained gel of a DARTS experiment. (**D**) List of STIRUR 41 putative protein partners. For each DARTS, the fold change is reported as a percentage, in respect of the untreated and undigested sample vs. the untreated and digested one.

In previous studies, STIRUR 41 (Figure 1A) has been characterized as a chemically stable pyrazole derivative with a good grade of lipophilicity and a theoretical clogP of 3.74, sufficient to permeate cell membranes. STIRUR 41 (tested as a racemic mixture) potently inhibited IL-8-induced chemotaxis of human neutrophils [9]. Among chemokines, IL-8 promotes the migration of neutrophils to the inflammation site, and cancer cells, to ease the metastatic process. In addition, STIRUR 41 was able to counteract high-risk-NB recurrence, blocking inflammation and angiogenesis, two mechanisms strongly involved in cancer progression and metastatization. In particular, the compound massively reduced the migratory ability of HTLA-230 cells, characterized by *MYCN* amplification, resulting in less effectiveness towards neuroblastoma ACN cells lacking this mutation [10]. In detail, STIRUR 41 treatment did not affect the viability and tumorigenicity of ACN and HTLA-230 cells, did not induce changes in p38MAPK and phosphop38MAPK levels of ACN and HTLA-230 cells, but it was able to counteract the HTLA-230 cells ability to form capillary-like structures [10].

Remarkably, the molecular mechanism at the basis of STIRUR 41 biological activities has not been investigated, and here we have applied a functional proteomics platform to disclose its most affine target protein(s) to explore its mechanism on HTLA-230.

Indeed, current advances in joining omics approaches with biochemical strategies are pointing to unveiling the alteration of cellular pathways exerted by small molecules [11]. Chemo-functional proteomics allows to detect interactions of a bioactive compound with its protein partners, mainly using thermal stability-based methods or enhanced resistance to limited proteolysis [12,13]. Indeed, small molecules binding to target proteins vary their tridimensional and conformational structure, mainly altering their exposure to proteases, inducing a stabilization of the folded state: drug affinity-responsive target stability (DARTS) displays the decrease in target protein(s) protease cuts upon the direct interaction with the bioactive compound. Likewise, limited proteolysis-coupled mass spectrometry (LiP-MS) quantitatively defines the target regions which are more affected by the interaction being thus involved, directly or via long-range conformational shifts, in the binding [14,15,16,17,18].

In this work, we applied our consolidated DARTS and LiP-MS platform [19,20,21] to study the interactome of STIRUR 41 racemate on HTLA-230, identifying Ubiquitin specific protease 7 (USP-7) as STIRUR 41 main target. Then, cross-validation of the mass spectrometry-based results has been achieved using immunoblotting. Docking simulations were also carried out to predict the binding mode of STIRUR 41 with USP-7. Moreover, STIRUR 41 inhibitory potential on USP-7 enzymatic activity and protein expression was confirmed through in vitro and in-cell assays.

## 2. Results

### 2.1. STIRUR 41 HTLA-230 Internalization Profile

The internalization and putative hydrolysis of STIRUR 41 have been investigated by liquid chromatography-multiple reaction monitoring-MS (LC-MRM-MS) by incubating HTLA-230 cells with the small molecule at the non-cytotoxic concentration of 100 µM as determined by MTT assays in Figure S1. In particular, STIRUR 41 internalization was evaluated by quantifying both the residual molecule in the cell media and the one in cell lysates, after 24 and 48 h of incubation. As shown in Figure 1B, the molecule straightforwardly permeates HTLA-230 cells, decreasing its amount in the cell media and increasing its internalization during the incubation times, up to around 80% after 48 h. Contextually, the putative formation of STIRUR 41 carboxylic acid derivative has been monitored: no signal was detected because no hydrolysis occurred at the ethyl ester of pyrazole after the same experimental times.

### 2.2. Identification of STIRUR 41 Protein Partner via DARTS-Based Experiments

DARTS is based on the principle that when, in a pseudo-physiological environment, a small molecule recognizes its specific protein target, it produces a complex, which has a more compacted tridimensional structure in respect of the target itself, and thus has a lower sensitivity to enzymatic proteolysis. Our optimized DARTS experiment is focused on the SDS-PAGE comparison of a cellular lysate, pre-treated or not with the small molecule, upon incubation with the low-specificity protease subtilisin under controlled conditions. In such a way, it is possible to monitor the altered proteins resistance to the enzymatic hydrolysis due to the small molecule shelter effect in a concentration-dependent fashion. Then, the target protein(s) are unambiguously identified through bottom-up proteomic approaches using in situ digestion, nano-UHPLC-MS/MS experiments, and bioinformatic tools.

In our experiments, HTLA-230 cells were lysed in mild non-denaturing conditions, and the obtained proteins were treated with increasing amounts of STIRUR 41 and then subjected to subtilisin limited proteolysis. Then, all the aliquots were submitted to 1D-SDS-PAGE separation and revealed by Coomassie blue staining (Figure 1C). Gel lanes of the treated lysates were cut in even pieces along the molecular weight marker until 30 kDa, digested, and submitted to a nano-UHPLC-MS/MS analysis followed by Proteome Discoverer search to reach proteins identification and label-free quantification. For each identified protein, a fold change is obtained, representing the ratio of the protein intensity in the STIRUR 41 treated sample vs. the untreated one at the same subtilisin amount. Several putative targets belonging to different biological pathways were identified at least in two out of three experiments (Figure 1D). Among them, we focalized our attention on the proteins involved in neuroblastoma development, namely, Ubiquitin specific protease 7 (i.e., USP-7), Nuclear RNA export factor 1 (i.e., NXF1), and Pescadillo homolog (i.e., PES1) [22,23,24].

**Figure 2:**
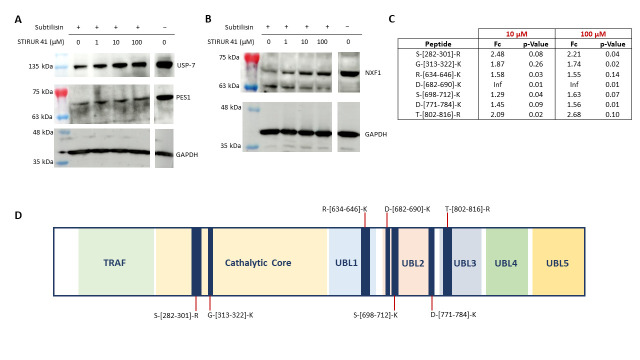
Validation of DARTS results for USP-7, PES1 (**A**), and NXF1 (**B**). (**C**) Peptides mapping for USP-7 regions involved in the binding with STIRUR 41, reported with the corresponding fold changes and *p*-Values. (**D**) USP-7 2D representation, showing the protein’s different domains in different colors and STIRUR 41 protected peptides as blue bars.

The direct interaction between these proteins and STIRUR 41 was evaluated by Western blotting, submitting all DARTS samples to an anti-USP-7, anti-PES, and anti-NXF1 antibody reaction. Comparing the intensity signals reported in the blots (Figure 2A,B) and in the densitometric analysis histograms in Figure S2, USP-7 results as the most reliable STIRUR 41 partner, since it increases its amount accordingly with the molecule concentrations, being more sheltered from proteolysis in respect of both PES1 and NXF1.

### 2.3. Ubiquitin Carboxyl-Terminal Hydrolase 7

Ubiquitin carboxyl-terminal hydrolase 7 (USP-7) is a monomeric cysteine protease that removes ubiquitin and saves substrate proteins from proteasomal degradation [25]. This protein is composed of three main domains: the N-terminal (amino acids 53–206), the central catalytic domain (amino acids 208–560), and the ubiquitin-like domain (UBL1-5, amino acids 560–1102) at the C-terminal end [26]. The catalytic domain, characterized by the presence of the C223-H464-D481 triad, has a binding pocket that catalyzes the hydrolysis of the isopeptide bond between ubiquitin and its substrates [27]. USP-7 has an unusual enzymatic mechanism in which the low intrinsic activity of the catalytic domain increases when its C-terminal domain folds, approaching the active site [28].

Aberrant activation or overexpression of USP-7 might promote oncogenesis, making this protein a promising target for cancer treatment [29]. Indeed, USP-7 was found to be highly expressed in a variety of malignant tumors, including myeloma [30], prostate cancer [26], hepatocellular cancer [31], ovarian cancer [32], and glioma [33]. In addition, increased USP-7 expression generally indicates a poor tumor prognosis. For all these reasons, USP-7 is a marker of tumor prognosis and a potential target for anti-tumor therapy [34].

### 2.4. Identification of USP-7 Regions Involved in STIRUR 41 Binding via Targeted-LiP-MRM

For a deeper characterization of STIRUR, 41 binding with USP-7, a targeted limited proteolysis (t-LiP) experiment coupled with Multiple Reaction Monitoring (MRM) MS has been optimized to pinpoint USP-7 regions involved, directly or not, in the binding with the small molecule. Indeed, these regions are more resistant to limited proteolysis with an unspecific protease, such as subtilisin, as evaluable after an additional extensive tryptic digestion: tryptic peptides should be present to a greater extent in those regions, which were protected from subtilisin by STIRUR 41, in a concentration-dependent trend.

A pilot experiment analyzing the tryptic digest of an HTLA-230 lysate has been carried out to identify USP-7 peptides coupling the signals of their precursor ions (revealed by Q1) to their best daughter ions (revealed by Q3), assigning the retention time to each peptide (Figure S3A). Thus, a 62% coverage of the USP-7 sequence has been obtained (Figure S3B). Then, HTLA 230 lysate aliquots incubated or not with STIRUR 41 were submitted to subtilisin limited proteolysis in native conditions, followed by a tryptic digestion in denaturing settings: the intensity of the tryptic peptides was then compared at different STIRUR 41 concentrations (10 and 100 µM) with the untreated sample, to select those regions influenced by the small molecule.

For each peptide, both the fold change (Fc) and the *p*-Value were calculated. The fold change is given by the ratio of the peak area relative to the STIRUR 41 treatment and the peak area of the respective control peptide (considering the average areas of three replicates). Only the fold changes ˃1.5 with *p*-Value ≤ 0.1 were considered to define altered peptides, and seven peptides were identified as changing upon STIRUR 41 treatment (Figure 2C). These peptides are also represented on the 2-dimensional sequence of USP-7, shown in panel D of Figure 2. It can be appreciated that two peptides are located in the catalytic site, while the other five are in the UBL1/2/3 domains of the C-terminal end, which is also important for de-ubiquitination activity. It is interesting that USP-7 full activity requires the C-terminal Ub-like domains to fold back onto the catalytic one, allowing the remodeling of the active site to a catalytically competent state.

Thus, encouraged by these results, we moved to an in silico approach to corroborate our LiP data.

### 2.5. Docking Simulations

To further characterize the interaction between STIRUR 41 and USP-7, two docking simulations were carried out: the first on the N-terminal bearing catalytic domain (residues 207–563; PDB code: 5N9T) [35] and the second on the C-terminal domain (residues 560–1102; PDB code: 5C56) [36]. Noteworthy, both regions were found to be involved in protein-ligand interaction in the t-LiP-MRM experiments.

**Figure 3:**
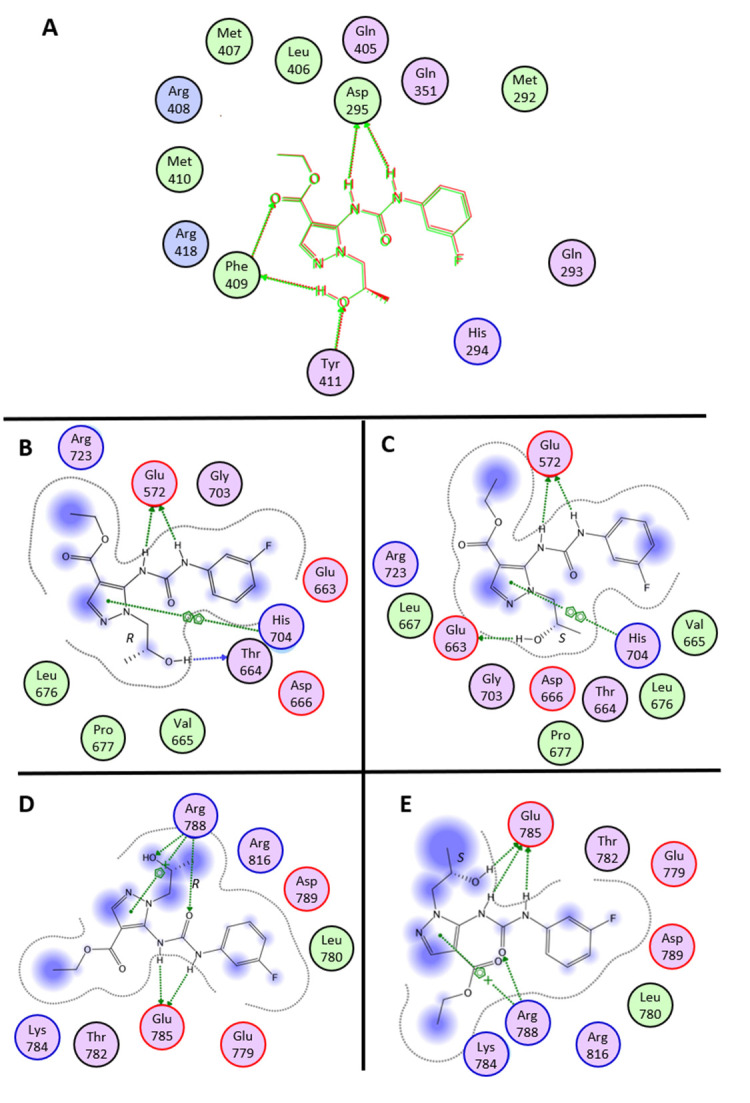
(**A**) Ligplot of the superposition of STIRUR 41 enantiomers in N-terminal USP-7 catalytic site. (**B**) Ligplot of the interaction between (R)-STIRUR 41 and USP-7 (site 2). (**C**) Ligplot of the interaction between (S)-STIRUR 41 and USP-7 (site 2). (**D**) Ligplot of the interaction between (R)-STIRUR 41 and USP-7 (site 4). (**E**) Ligplot of the interaction between (S)-STIRUR 41 and USP-7 (site 4).

According to the calculations, (R)-STIRUR 41 and (S)-STIRUR 41 would assume a similar orientation in the catalytic pocket of the N-terminal portion (Table S1). The complexes are mainly stabilized by a network of hydrogen bonds involving the Asp295 side chain and Phe409 and Tyr411 main chain atoms (Figure 3A). Interestingly, two H-bonds were recognized with Asp295: an alteration in this region has also been detected by t-LiP-MRM. The complexes would be further stabilized by Van der Waals interactions occurring between the pyrazole substructure and the Met407 side chain. The 3-fluorophenyl ring would interact with Met292, Gln293, and His294 main chains, whereas the ester functionality would be in contact with Leu406, Arg408, and Asn418. Consistently with similar interaction networks, the calculated Ki values for the two complexes are comparable (i.e., Ki_(R)-STIRUR 41/USP-7_ = 111.86 µM; Ki_(S)-STIRUR 41/USP-7_ = 109.41 µM).

To identify the putative binding site of STIRUR 41 in the USP-7 C-terminal domain, a site finder search (MOE 2009.10 software) was performed. Two binding pockets (namely, site 2 and site 4; Figure S6) were selected on the basis of their size (site 2: 124 Å^2^; site 4: 102 Å^2^) and their proximity to the peptide residues identified in the t-LiP-MRM experiments. Both STIRUR 41 enantiomers were modeled and docked in site 2 and site 4 (Table S1).

(R)-STIRUR 41 and (S)-STIRUR 41 would assume a similar orientation in the site 2 binding site. Both complexes would be mainly stabilized by two hydrogen bonds involving the urea NH group and Glu572 side chain and an arene-arene interaction between the pyrazole substructure and His704 side chain. Moreover, the (R)-STIRUR 41 hydroxyl group would be hydrogen-bonded with Tyr664 backbone carbonyl, whereas in the (S)-STIRUR 41/USP-7 complex, a similar interaction would occur with the Glu663 side chain (Figure 3B,C). Consistently with similar interaction networks, the calculated Ki values for the two complexes are comparable (i.e., Ki_(R)-STIRUR 41/USP-7_ = 183.72 µM; Ki_(S)-STIRUR 41/USP-7_ = 171.79 µM).

In site 4, the bioactive conformations calculated for the two STIRUR 41 enantiomers will superimpose with the 3-fluorophenyl moiety pointing towards the protein core and the pyrazole substructure projecting towards the solvent. In detail, (R)-STIRUR 41 would extensively interact with the Arg788 side chain through the formation of two hydrogen bonds with the urea carbonyl group and hydroxyl functionality and a cation-π interaction with the pyrazole nucleus. Additionally, the urea NH groups would form two hydrogen bonds with the Glu785 side chain, thus providing further stabilization to the complex (Figure 3D). Similarly, in the (S)-STIRUR 41/USP-7 docking complex, Glu785 and Arg788 side chains would be extensively involved in the formation of stabilizing interactions with the ligands (Figure 3E). The Ki values calculated for (R)-STIRUR 41/USP-7 and (S)-STIRUR 41/USP-7 docking complex were 965.24 µM and 919.62 µM, respectively.

Thus, docking simulations pointed towards a higher affinity interaction of both STIRUR 41 enantiomers with the catalytic site, suggesting a possible small molecule mediated inhibition of the enzyme activity, as corroborated by the secondary most affine binding site found as site 2. Indeed, UBL1 and two domains are similarly responsible for USP-7 activation.

### 2.6. Evaluation of STIRUR 41 Effects on USP-7 Enzymatic Activity and Expression Profile

**Figure 4:**
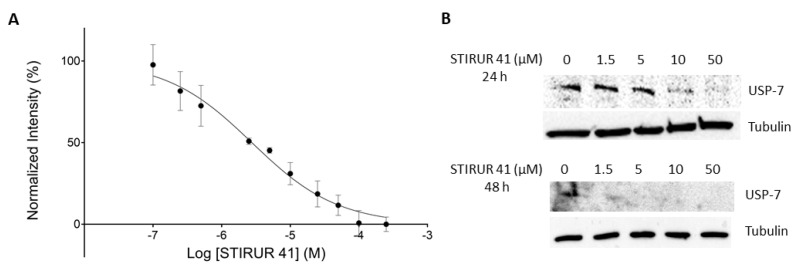
(**A**) IC_50_ curve of USP-7 inhibition by STIRUR 41, obtained through GraphPad Prism 7. Each data point is represented with the relative standard deviations. (**B**) Western Blot showing USP-7 protein levels in HTLA-230 cells treated (or not) with increasing amounts of STIRUR 41 at 24 h and 48 h. Tubulin was exploited as a loading normalizer.

The inhibition efficiency of STIRUR 41 on USP-7 has been measured through a specific fluorescence kit that employs Ubiquitin-AMC as substrate: when the substrate is hydrolyzed by recombinant USP-7, the fluorescence signal increases. Thus, USP-7 was incubated with different amounts of STIRUR 41, then Ubiquitin-AMC was added, and the fluorescence was measured: STIRUR 41 caused a reduction in fluorescence intensity, resulting in an IC_50_ of 2.77 ± 0.56 µM (R^2^ = 0.947) (Figure 4A).

Interestingly, a close structural analog of STIRUR 41 called GeGe-3, whose interactome has been disclosed by our group and that did not show any affinity for de-ubiquitinase enzymes [20,37], was fully inactive towards recombinant USP-7 in the same assay, underlying STIRUR 41 modulation specificity (Figure S4A). As a positive control of the inhibition event, the well-known USP-7 inhibitor P02277 was tested (Figure S4B).

Subsequently, to determine whether STIRUR 41 could also modulate USP-7 expression, HTLA-230 cells were treated with increasing amounts of the small molecule (i.e., 1.5, 5, 10, and 50 µM) for 24 and 48 h. Then, cells were lysed, and the samples were submitted to Western Blotting. As reported in Figure 4B, 10 and 50 µM STIRUR 41 induced a remarkable decrease in USP-7 protein levels compared to the untreated cells at 24 h and, accordingly with the internalization behavior reported above, at 48 h also the lowest STIRUR 41 concentration reduced USP-7 expression. As a reference compound, P02277 was also tested (Figure S5).

## 3. Discussion

The biological effects of STIRUR 41, an inhibitor of IL8-induced neutrophil chemotaxis, were tested on ACN and HTLA-230 neuroblastoma cells, the latter being characterized by high plasticity and aggressiveness due to *MYCN* gene amplification [10]. The published data showed that the molecule is not cytotoxic, nor it alters clonogenic capacity on both cell lines. Furthermore, STIRUR 41 showed anti-migratory and anti-angiogenic activity on HTLA-230 cells [10], and, in this paper, we moved to disclose its interactome.

Even though nowadays it is common knowledge that small bioactive molecules have the capability of modulating more than one single pathway via the interaction with multiple receptors; here, one of the possible STIRUR 41 mechanisms of action has been unveiled. Its interaction with three putative partners has emerged from DARTS experiments, namely, USP-7, NXF1, and PES1, and further immunoblotting demonstrated that the most affine partner was USP-7. The subsequent t-LiP-MRM data showed that STIRUR 41 induced conformational changes in both the USP-7 catalytic site and in the UBL1/2 domain of the C-terminal end; both pieces of evidence were corroborated by docking simulations on STIRUR 41 enantiomers. According to the calculated Ki values, STIRUR 41 would preferentially bind in the catalytic pocket and in C-terminal Site 2. In vitro activity assays further confirmed the proteomics data, showing how the interaction with STIRUR 41 inhibited USP-7 activity in a concentration-dependent fashion and with an IC_50_ in the low-micromolar range. With the aim of investigating STIRUR 41 potential to modulate USP-7 expression in living cells, we also examined USP-7 protein levels in HTLA-230 cells treated with the small molecule. As expected, STIRUR 41 was able to reduce the protein expression at both 10 µM and 50 µM tested concentration.

In conclusion, since the downregulation of USP-7 activity and of its expression seems to be a crucial step in the modulation of *MYCN* amplified neuroblastoma cells, the discovery of STIRUR 41 effect on this enzyme paves the way for considering this chemical scaffold as a starting point for the development of new molecules active in the treatment of nervous system cancer.

## 4. Materials and Methods

STIRUR 41 racemate was synthesized and characterized as previously reported [9].

### 4.1. 3-(4,5-Dimethylthiazol-2-yl)-2,5-diphenyltetrazolium Bromide (MTT) Assay

HTLA-230 cell viability was calculated through the MTT assay as previously reported [38]. Thus, cells were treated for 24 and 48 h with STIRUR 41 at 1, 5, 10, 25, 50, and 100 µM and, at the end of such experimental times, 25 μL of a 5 mg/mL MTT stock solution was added to each well in 100 μL of the medium, for 3 h at 37 °C. Next, cells were lysed in DMSO (100 μL/well). The optical density (OD) of each well was measured with a microplate spectrophotometer (Titertek Multiskan MCC/340, San Bruno, CA, USA) equipped with a 550 nm filter. The viability of cells in response to treatment was calculated as: % viable cells = [OD treated cells/OD control] × 100.

### 4.2. STIRUR 41 Metabolic Profile by LC-MS-MRM Analysis

Optimization of the MS spectrum and MS/MS fragmentation has been obtained for STIRUR 41 by direct infusion on a QTRAP 6500 (AB Sciex LLC, Framingham, MA, USA). Several compound solutions were prepared at concentrations ranging from 10 nM to 200 µM for the calibration curve. Each sample was loaded onto the LC-20A Shimadzu UHPLC–MS system coupled to the QTRAP 6500. Chromatographic separation was carried out on a Luna Omega Polar C18 (50 × 2.1 mm, 1.6 μm, 100 Å, Phenomenex, Torrance, CA, USA) by means of a linear gradient from 10–95% from H_2_O to CH_3_CN with 0.1% formic acid (FA) in 10 min and at 400 µL/min.

Mass spectra were acquired in MRM positive polarity using DP (volts) of 80, EP (volts) of 11, CE (volts) of 22, CXP (volts) of 20 and monitoring the following transitions: STIRUR 41 @ 351 to 305; @ 351 to 214; @ 351 to 196; @ 351 to 168; @ 351 to 150; for its carboxylic putative derivative @ 323 to 305; @ 323 to 186; @ 323 to 168; @ 323 to 150; @ 323 to 323.

HTLA-230 cells were incubated with 100 µM STIRUR 41 or control vehicle (DMSO, 0.01% vol/vol) for 0, 24, and 48 h in triplicate, and the media and the cell pellets were recovered separately. The pellets were extensively washed, then lysed using M-PER (Mammalian Protein Extraction Reagent, Thermo Scientific, Waltham, MA, USA) supplied with a protease inhibitor cocktail (GeneSpin, Milan, Italy) and proteins were quantified by Bradford assay. Then, equal proteins amount for the cell lysates and equal volumes for the media were precipitated by ice-cold acetone, and the supernatants were dried and re-suspended in an opportune volume of 80% H_2_O, 20% CH_3_CN, 0.1% FA and loaded on the UHPLC–MS system as reported above.

### 4.3. DARTS-Based Experiments

HTLA-230 cell pellets were lysed in M-PER (Mammalian Protein Extraction Reagent, Thermo Scientific, Waltham, MA, USA) supplied with the protease inhibitor cocktail (GeneSpin, Milan, Italy). The lysate was centrifuged at 10,000× *g* for 10 min at 4 °C, and Bradford assay was used to determine the protein concentration of the obtained supernatant. Equal protein aliquots were incubated with DMSO as the vehicle or increasing amounts of STIRUR 41 (final concentrations of 1 μM, 10 μM, and 100 μM) for 1 h at room temperature. Samples were then submitted to limited proteolysis (1:1500 *w*/*w* subtilisin to proteins ratio) for 30 min (25 °C, 450 rpm). An aliquot of the untreated lysate was submitted to mock proteolysis. Subtilisin was then quenched with 1 mM phenylmetane-sulfonil-fluoride (PMSF, Sigma Aldrich-Merck, Milan, Italy). These DARTS experiments were performed in triplicate. Then, SDS PAGE runs and gel digestion were carried out as previously reported [20].

UHPLC-MS/MS analysis was performed by injecting 1 μL of each digest on an Orbitrap Q-Exactive Classic Mass Spectrometer coupled to an UltiMate 3000 Ultra-High-Pressure Liquid Chromatography system (ThermoFisher Scientific, Bremen, Germany), equipped with an EASY-Spray PepMAP^TM^ RSLC C18 column (3 μm, 100 Å, 75 μm × 50 cm, ThermoFisher Scientific, Bremen). Peptides elution occurred at a flow rate of 300 nL/min with the following gradient: 1 min at 3% B, 1 min to 40 min to 28% B, 40 min to 41 min to 70% B, 41 min to 49 min at 70% B and 50 min back to 3% B until 60 min (A: 95% H_2_O, 5% CH_3_CN, 0.1% AcOH; B: 95% CH_3_CN, 5% H_2_O, 0.1% AcOH). The mass spectrometer was operated in data-dependent acquisition mode, acquiring full scan MS spectra with a scan range of 375–1500 *m*/*z*, a full-scan automatic gain control (AGC) target 3 × 10^6^ at 70,000 resolution, and a maximum injection time of 50 ms. MS2 spectra were generated for up to 8 precursors, with a normalized collision energy of 28%, and the fragments were acquired at a resolution of 17,500, with an AGC target of 1 × 10^5^ and a maximum injection time of 80 ms.

Proteome Discoverer (version 2.4.1.15) was then exploited for both proteins’ identification and quantification. MSPepSearch was used to perform a spectral library search (NIST Human Orbitrap HCD Library, 1,127,970 spectra, September 2016) with an MS1 mass tolerance of 10 ppm and an MS2 one of 0.02 Da. FDR was set to 1% (strict) and 5% (relaxed). Spectra were also searched by Sequest against a reviewed *Homo sapiens* database (SwissProt, February 2022, 20,607 entries) using the following parameters: trypsin digestion; maximum of two missed cleavages; cysteine carboxyamido-methylation as fixed modification; methionine oxidization, protein N-terminal acetylation, and/or demethylation as variable modifications.

Label-free quantification was performed using both unique and razor peptides for protein abundance calculation, and a pairwise ratio-based approach was used to evaluate STRUR41 vs. ctrl proteins abundances ratios. For each calculated ratio, a background-based t-test was performed.

### 4.4. DARTS Immunoblotting Validation

Immunoblotting analyses were carried out by submitting 20 μg of the DARTS protein mixtures to 1D-SDS-PAGE, then transferring the gels onto nitrocellulose membranes through the Trans-Blot Turbo Transfer System (Bio-Rad, Hercules, CA, USA) for 15 min at 2.5 A (up to 25 V). The membranes were blocked for 1 h at room temperature in a 5% w/vol non-fat dried milk solution and then incubated overnight at 4 °C with the following primary antibodies: anti-USP-7 (SC-137008, Santa Cruz Biotechnology, Dallas, TX, USA) 1:250 vol/vol, anti-PES (SC-166300, Santa Cruz Biotechnology, Dallas, TX, USA) 1:500 vol/vol, anti-NXF1 (SC-32319, Santa Cruz Biotechnology, Dallas, TX, USA) 1:500 vol/vol, then mouse peroxidase-conjugated secondary antibody was added (1:500 vol/vol Thermo-Scientific, Bremen, Germany). Membranes were also hybridized with an anti-GAPDH primary antibody (SC-47724, Santa Cruz Biotechnology, Dallas, TX, USA) 1:2000 vol/vol) for loading normalization purposes.

### 4.5. USP-7 MRM Method Building

USP-7 (UniProt Accession: Q93009) tryptic peptides were selected through the data resource Peptide Atlas (https://db.systemsbiology.net/sbeams/cgi/PeptideAtlas, accessed on 1 March 2022) on its Human build and queried into the complete Human SRM Atlas (https://db.systemsbiology.net/sbeams/cgi/PeptideAtlas/GetTransitions, accessed on 1 March 2022) to get their best daughter ions. Thus, methods listing USP-7 peptides and their three best MS/MS fragments were obtained and tested onto an HTLA-230 tryptic digest. An HTLA-230 cell lysate was submitted to an in-solution digestion protocol, then desalted and analyzed through UHPLC-MRM-MS as previously reported [20].

### 4.6. Targeted-LiP-MRM

HTLA-230 cells proteome aliquots, obtained as previously reported, were incubated with STIRUR 41 (10 and 100 μM final concentrations) for 1 h at room temperature. Samples were then submitted to limited proteolysis with 1:1500 and 1:2500 (*w*/*w*) subtilisin to proteins ratios, then subtilisin was quenched with PMSF (1 mM), and all of the samples were denatured and digested with trypsin/LysC. Peptides mixtures were then desalted and injected into the UHPLC-ESI-MRM system, as already reported [20] and analyzed through the previously optimized MRM methods. The area of each USP-7 tryptic peptide peak was measured using the Analyst Software 1.6.2 (AB Sciex LLC, Framingham, MA, USA). Each sample was analyzed in triplicate. STIRUR41-treated vs. untreated tryptic peptides areas ratios were calculated from the averages of the areas of each peptide, and a t-test was performed to calculate the associated *p*-Values.

### 4.7. Docking Simulations

The molecular structures of compounds (*R*)-STIRUR 41 and its enantiomer (*S*)-STIRUR 41 were built by MOE2009.10 (builder module), parameterized by MMFF94x force field, and their docking poses within USP-7 N-terminal and C-terminal domains were calculated by Autodock 4.2 [39].

All water molecules and ligand coordinates were removed from the crystal structure of the N-terminal USP-7 domain (PDB code: 5N9T) [35]. Polar hydrogen and Gasteiger-Huckel charges were then added, and a 60 × 60 × 60 Å grid (grid spacing 0.375 Å) was centered in the binding site, as defined in the crystal structure. Electrostatic and affinity maps for each atom type of the ligand were calculated. The docking search was performed over 100 conformers using the Genetic Algorithm Local Search protocol as implemented in Autodock (population size: 50; rate of gene mutation: 0.02; rate of crossover: 0.8). The docking poses were clustered (rmsd: 2.0 Å) and the best conformation of the low energy highest populated cluster was selected as the binding conformation.

Similarly, after the removal of the water molecules from the coordinates of the USP-7 C-terminal domain (PDB code: 5C56) [36], polar hydrogens and Gasteiger-Huckel charges were added. A site finder search was carried out by MOE2009.10 (site finder module; probe radius 1 = 1.4 Å; probe radius 2 = 1.8 Å; connection distance = 2.5 Å) to identify putative binding sites in the structure. Sites 2 and 4 were identified and selected on the basis of their sizes and proximity to the region identified by t-LiP experiments. Two 80 × 80 × 80 Å grids (grid spacing 0.375 Å) were centered in the two putative binding sites and electrostatic and affinity maps for each atom type of the ligand were calculated. Two docking searches were performed independently on the two sites adopting a Genetic Algorithm Local Search protocol as implemented in Autodock (population size: 50; rate of gene mutation: 0.02; rate of crossover: 0.8; number of runs: 100). The analysis and selection of the docking poses were carried out according to the above-mentioned protocol. Models analysis was carried out using the CCP4 program suite [40]. The calculations were run on a Linux PC (Intel® processor Core™ i7-2600 CPU@3.40 GHz; Santa Clara, CA, USA).

### 4.8. Ub-AMC Assay

In vitro enzymatic inhibition was measured using the USP-7 inhibitor screening assay kit (catalog #79256, BPS Bioscience, San Diego, CA, USA) according to the manufacturer’s instructions. Briefly, STIRUR 41 stock solutions were prepared in DMSO, then diluted 10-fold to obtain the working solutions. Then, these solutions and the vehicle control were added to the black 384-wells plate together with the USP-7 enzyme, previously diluted as required by the manufacturer, and incubated at r.t. for 30 min. Then, the Ub-AMC substrate was diluted 400-fold and added to the plate, followed by incubation at r.t. for 30 min. The final DMSO amount in each well was 1%. The fluorescence signal (excitation = 350 nm, emission = 460 nm) was recorded using the EnSight microplate reader (PerkinElmer, Waltham, MA, USA). IC_50_ was calculated by subtracting Ub-AMC background from each value and using GraphPad Prism 7 software.

### 4.9. Evaluation of USP-7 Protein Expression Levels Modulation by STIRUR 41

HTLA-230 cells (5 × 10^4^ cells/well) were seeded in a 24-well plate and, after 24 h, were treated with STIRUR 41 at 1.5, 5, 10, and 50 µM for 24 and 48 h. Total extracted proteins were examined by SDS-PAGE and Western Blotting, as previously described [41]. Membranes were incubated overnight at 4 °C with a primary monoclonal antibody against USP-7 (1:500 vol/vol, Santa Cruz Biotechnologies, Dallas, TX, USA) and tubulin (1:1000 vol/vol; Sigma-Aldrich; St. Louis, MO, USA), then at room temperature with an appropriate secondary anti-mouse antibody (1:5000 vol/vol; Sigma-Aldrich; St. Louis, MO, USA). Immunoreactive protein bands were detected using enhanced chemiluminescence reagents (ECL; Amersham biosciences; Little Chalfont, UK); the blots were exposed and analyzed by means of LAS4000 (GE Healthcare Life Sciences; Buckinghamshire, UK).

## Data Availability

Data will be available on request.

## Supplementary Materials

The supporting information can be downloaded at: https://www.mdpi.com/article/10.3390/ijms24119200/s1.

## References

[1] Maris J.M., Hogarty M.D., Bagatell R., Cohn S.L. (2007). Neuroblastoma. Lancet.

[2] Friedman G.K., Castleberry R.P. (2007). Changing trends of research and treatment in infant neuroblastoma. Pediatr. Blood Cancer.

[3] Pistoia V., Bianchi G., Borgonovo G., Raffaghello L. (2011). Cytokines in neuroblastoma: From pathogenesis to treatment. Immunotherapy.

[4] Huang M., Weiss W.A. (2013). Neuroblastoma and MYCN. Cold Spring Harb. Perspect. Med..

[5] Ruiz-Pérez M.V., Henley A.B., Arsenian-Henriksson M. (2017). The MYCN Protein in Health and Disease. Genes.

[6] Hendrix M.J.C., Seftor E.A., Hess A.R., Seftor R.E.B. (2003). Vasculogenic mimicry and tumour-cell plasticity: Lessons from melanoma. Nat. Rev. Cancer.

[7] Ribatti D., Marimpietri D., Pastorino F., Brignole C., Nico B., Vacca A., Ponzoni M. (2004). Angiogenesis in neuroblastoma. Ann. N. Y. Acad. Sci..

[8] Tavana O., Li D., Dai C., Lopez G., Banerjee D., Kon N., Chen C., Califano A., Yamashiro D.J., Sun H. (2016). HAUSP deubiquitinates and stabilizes N-Myc in neuroblastoma. Nat. Med..

[9] Bruno O., Brullo C., Bondavalli F., Schenone S., Spisani S., Falzarano M.S., Varani K., Barocelli E., Ballabeni V., Giorgio C. (2009). 1-Methyl and 1-(2-hydroxyalkyl)-5-(3-alkyl/cycloalkyl/phenyl/naphthylureido)-1H-pyrazole-4-carboxylic acid ethyl esters as potent human neutrophil chemotaxis inhibitors. Bioorg. Med. Chem..

[10] Marengo B., Meta E., Brullo C., De Ciucis C., Colla R., Speciale A., Garbarino O., Bruno O., Domenicotti C. (2023). Correction: Biological evaluation of pyrazolyl-urea and dihydro-imidazo-pyrazolyl-urea derivatives as potential anti-angiogenetic agents in the treatment of neuroblastoma. Oncotarget.

[11] Meissner F., Geddes-McAlister J., Mann M., Bantscheff M. (2022). The emerging role of mass spectrometry-based proteomics in drug discovery. Nat. Rev. Drug Discov..

[12] Lomenick B., Hao R., Jonai N., Chin R.M., Aghajan M., Warburton S., Wang J., Wu R.P., Gomez F., Loo J.A. (2009). Target identification using drug affinity responsive target stability (DARTS). Proc. Natl. Acad. Sci. USA.

[13] Feng Y., De Franceschi G., Kahraman A., Soste M., Melnik A., Boersema P.J., de Laureto P.P., Nikolaev Y., Oliveira A.P., Picotti P. (2014). Global analysis of protein structural changes in complex proteomes. Nat. Biotechnol..

[14] Morretta E., Esposito R., Festa C., Riccio R., Casapullo A., Monti M.C. (2017). Discovering the biological target of 5-epi-sinuleptolide using a combination of proteomic approaches. Mar. Drugs.

[15] Morretta E., Tosco A., Festa C., Mozzicafreddo M., Monti M.C., Casapullo A. (2020). Crellastatin A, a PARP-1 Inhibitor Discovered by Complementary Proteomic Approaches. ChemMedChem.

[16] Del Gaudio F., Pollastro F., Mozzicafreddo M., Riccio R., Minassi A., Monti M.C. (2018). Chemoproteomic fishing identifies arzanol as a positive modulator of brain glycogen phosphorylase. Chem. Commun..

[17] Schopper S., Kahraman A., Leuenberger P., Feng Y., Piazza I., Müller O., Boersema P.J., Picotti P. (2017). Measuring protein structural changes on a proteome-wide scale using limited proteolysis-coupled mass spectrometry. Nat. Protoc..

[18] Hwang H.-Y., Kim T.Y., Szász M.A., Dome B., Malm J., Marko-Varga G., Kwon H.J. (2020). Profiling the Protein Targets of Unmodified Bio-Active Molecules with Drug Affinity Responsive Target Stability and Liquid Chromatography/Tandem Mass Spectrometry. Proteomics.

[19] Ceccacci S., Deitersen J., Mozzicafreddo M., Morretta E., Proksch P., Wesselborg S., Stork B., Monti M.C. (2020). Carbamoyl-phosphate synthase 1 as a novel target of phomoxanthone a, a bioactive fungal metabolite. Biomolecules.

[20] Morretta E., Belvedere R., Petrella A., Spallarossa A., Rapetti F., Bruno O., Brullo C., Monti M.C. (2021). Novel insights on the molecular mechanism of action of the anti-angiogenic pyrazolyl-urea GeGe-3 by functional proteomics. Bioorg. Chem..

[21] Morretta E., Sidibè A., Spallarossa A., Petrella A., Meta E., Bruno O., Monti M.C., Brullo C. (2021). Synthesis, functional proteomics and biological evaluation of new 5-pyrazolyl ureas as potential anti-angiogenic compounds. Eur. J. Med. Chem..

[22] Nakaguro M., Kiyonari S., Kishida S., Cao D., Murakami-Tonami Y., Ichikawa H., Takeuchi I., Nakamura S., Kadomatsu K. (2015). Nucleolar protein PES1 is a marker of neuroblastoma outcome and is associated with neuroblastoma differentiation. Cancer Sci..

[23] Malone C.F., Dharia N.V., Kugener G., Forman A.B., Rothberg M.V., Abdusamad M., Gonzalez A., Kuljanin M., Robichaud A.L., Conway A.S. (2021). Selective Modulation of a Pan-Essential Protein as a Therapeutic Strategy in Cancer. Cancer Discov..

[24] Fan Y.-H., Cheng J., Vasudevan S.A., Dou J., Zhang H., Patel R.H., Ma I.T., Rojas Y., Zhao Y., Yu Y. (2013). USP7 inhibitor P22077 inhibits neuroblastoma growth via inducing p53-mediated apoptosis. Cell Death Dis..

[25] Everett R.D., Meredith M., Orr A., Cross A., Kathoria M., Parkinson J. (1997). A novel ubiquitin-specific protease is dynamically associated with the PML nuclear domain and binds to a herpesvirus regulatory protein. EMBO J..

[26] Qi S.-M., Cheng G., Cheng X.-D., Xu Z., Xu B., Zhang W.-D., Qin J.-J. (2020). Targeting USP7-Mediated Deubiquitination of MDM2/MDMX-p53 Pathway for Cancer Therapy: Are We There Yet?. Front. Cell Dev. Biol..

[27] Harakandi C., Nininahazwe L., Xu H., Liu B., He C., Zheng Y.-C., Zhang H. (2021). Recent advances on the intervention sites targeting USP7-MDM2-p53 in cancer therapy. Bioorg. Chem..

[28] Kim R.Q., Geurink P.P., Mulder M.P.C., Fish A., Ekkebus R., El Oualid F., van Dijk W.J., van Dalen D., Ovaa H., van Ingen H. (2019). Kinetic analysis of multistep USP7 mechanism shows critical role for target protein in activity. Nat. Commun..

[29] Wang Z., Kang W., You Y., Pang J., Ren H., Suo Z., Liu H., Zheng Y. (2019). USP7: Novel Drug Target in Cancer Therapy. Front. Pharmacol..

[30] Chauhan D., Tian Z., Nicholson B., Kumar K.G.S., Zhou B., Carrasco R., McDermott J.L., Leach C.A., Fulcinniti M., Kodrasov M.P. (2012). A Small Molecule Inhibitor of Ubiquitin-Specific Protease-7 Induces Apoptosis in Multiple Myeloma Cells and Overcomes Bortezomib Resistance. Cancer Cell.

[31] Cai J., Shi G., Dong Z., Ke A., Ma H., Gao Q., Shen Z., Huang X., Chen H., Yu D. (2015). Ubiquitin-specific protease 7 accelerates p14ARF degradation by deubiquitinating thyroid hormone receptor-interacting protein 12 and promotes hepatocellular carcinoma progression. Hepatology.

[32] Yu N., Ma M. (2016). Ubiquitin-specific protease 7 expression is a prognostic factor in epithelial ovarian cancer and correlates with lymph node metastasis. OncoTargets Ther..

[33] Cheng C., Niu C., Yang Y., Wang Y., Lu M. (2013). Expression of HAUSP in gliomas correlates with disease progression and survival of patients. Oncol. Rep..

[34] Lu J., Zhao H., Yu C., Kang Y., Yang X. (2021). Targeting Ubiquitin-Specific Protease 7 (USP7) in Cancer: A New Insight to Overcome Drug Resistance. Front. Pharmacol..

[35] Gavory G., O’Dowd C.R., Helm M.D., Flasz J., Arkoudis E., Dossang A., Hughes C., Cassidy E., McClelland K., Odrzywol E. (2018). Discovery and characterization of highly potent and selective allosteric USP7 inhibitors. Nat. Chem. Biol..

[36] Cheng J., Li Z., Gong R., Fang J., Yang Y., Sun C., Yang H., Xu Y. (2015). Molecular mechanism for the substrate recognition of USP7. Protein Cell.

[37] Meta E., Imhof B.A., Ropraz P., Fish R.J., Brullo C., Bruno O., Sidibé A. (2017). The pyrazolyl-urea GeGe3 inhibits tumor angiogenesis and reveals dystrophia myotonica protein Kinase (DMPK)1 as a novel angiogenesis target. Oncotarget.

[38] Belvedere R., Novizio N., Eletto D., Porta A., Bagnulo A., Cerciello A., Di Maio U., Petrella A. (2021). The Procoagulant Activity of Emoxilane®: A New Appealing Therapeutic Use in Epistaxis of the Combination of Sodium Hyaluronate, Silver Salt, α-tocopherol and D-panthenol. Life.

[39] Morris G.M., Huey R., Lindstrom W., Sanner M.F., Belew R.K., Goodsell D.S., Olson A.J. (2009). AutoDock4 and AutoDockTools4: Automated docking with selective receptor flexibility. J. Comput. Chem..

[40] Winn M.D., Ballard C.C., Cowtan K.D., Dodson E.J., Emsley P., Evans P.R., Keegan R.M., Krissinel E.B., Leslie A.G.W., McCoy A. (2011). Overview of the CCP4 suite and current developments. Acta Crystallogr. D Biol. Crystallogr..

[41] Belvedere R., Novizio N., Pessolano E., Tosco A., Eletto D., Porta A., Campiglia P., Perretti M., Filippelli A., Petrella A. (2020). Heparan sulfate binds the extracellular Annexin A1 and blocks its effects on pancreatic cancer cells. Biochem. Pharmacol..

