# Supplementary material for: Targeting USP-7 by a Novel Fluorinated 5-Pyrazolyl-Urea Derivative
Source: Int J Mol Sci. 2023 May 24;24(11):9200. doi: 10.3390/ijms24119200 (PMC10252683; doi:10.3390/ijms24119200)
Supplement: Supplementary file 1 [file ijms-24-09200-s001.zip › ijms-2407609-supplementary.pdf]

Supplementary Materials

Targeting USP-7 by a novel fluorinated 5-pyrazolyl-urea derivative

Elva Morretta [1], Chiara Brullo [2], Raffaella Belvedere [1], Antonello Petrella [1], Andrea Spallarossa [2] and Maria Chiara Monti [1,*]

[1] Department of Pharmacy, University of Salerno, Via Giovanni Paolo II, 84084 Fisciano, Salerno, Italy; emorretta@unisa.it (E.M.), rbelvedere@unisa.it (R.B.), apetrella@unisa.it (A.P.)
[2] Department of Pharmacy, University of Genova, Viale Benedetto XV, 3, 16132 Genova, Italy; chiara.brullo@unige.it (C.B.), andrea.spallarossa@unige.it (A.S.)
* Correspondence: mcmonti@unisa.it (M.C.M.)

Figure S1: HTLA-230 cells viability evaluation.
HTLA3-230 cells viability was evaluated through MTT assay, treating the cells with increasing concentrations of STIRUR 41 (from 1 to 100 μM) for 24 and 48 hours. The obtained results are showed in the histogram, where each bar represents the mean Optical Density (i.e., OD) of 3 experiments reported with the relative standard deviation. As can be observed, none of STIRUR 41 concentrations induced cytotoxicity on the cell line at both the experimental times.

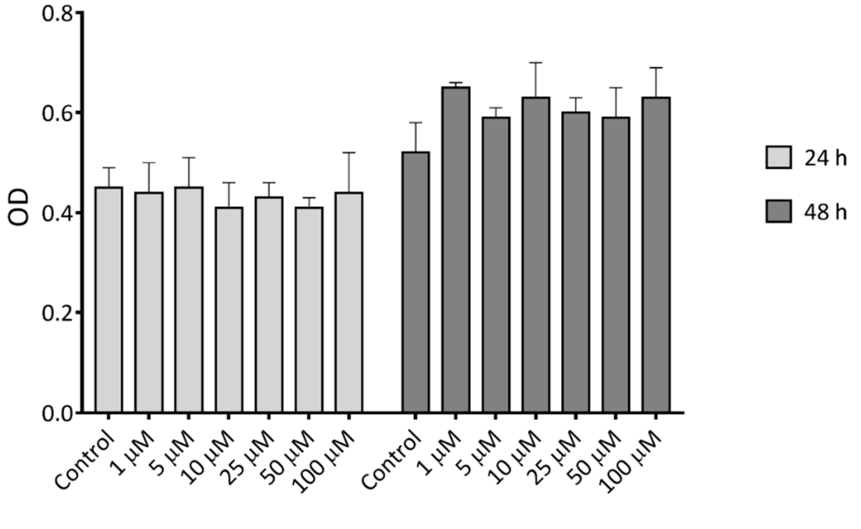

Figure S2: Western Blots Densitometric analysis.
Densitometric analysis of the Western Blots in Figure 2A and 2B, for USP-7 (A) PES1 (B) and NXF1 (C).

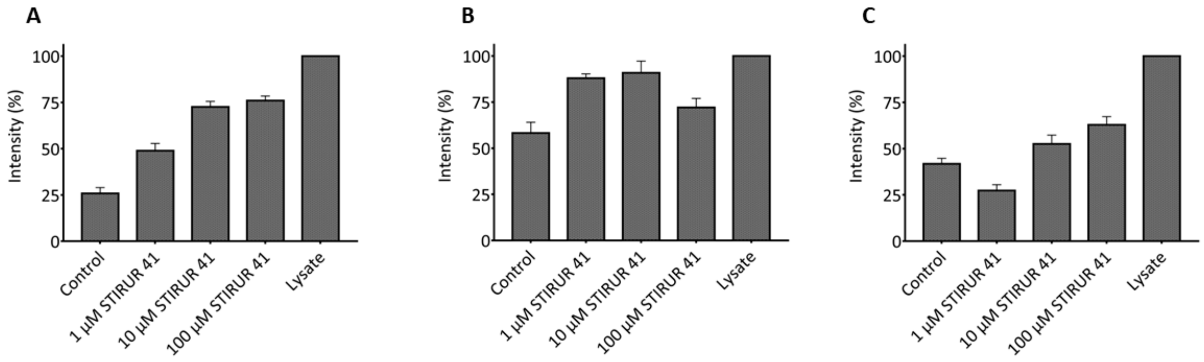

Figure S3: T-LiP-MRM pilot experiment.

(A) List of USP-7 tryptic peptides selected through *PeptideAtlas* and *SRMAtlas* and analyzed on HTLA-230 tryptic digest. Each peptide is reported with its precursor ion m/z value (i.e., Q1_mz), it best fragment ion m/z value (i.e., Q3_mz), and the related amino acids numbering (i.e., ID) and retention time. (B) USP-7 sequence shown with adjacent tryptic peptides in black and red for visualization purposes. Panel A peptides mapped on the protein sequence are highlighted in yellow (62% sequence coverage).

A

Q1_m/z	Q3_m/z	ID	RT (min)
373.20	433.25	D-[682-690]-K	2.45
373.21	333.18	A-[928-934]-K	10.96
388.56	694.40	D-[863-871]-R	4.88
389.45	216.12	E-[985-997]-K	6.69
413.21	653.37	D-[847-854]-R	6.77
453.72	565.23	D-[920-926]-K	4.75
462.74	219.11	F-[1040-1047]-R	3.16
476.72	263.10	F-[480-487]-R	4.49
559.77	342.20	D-[715-723]-R	6.99
612.30	274.12	C-[896-904]-R	4.91
618.80	259.09	E-[905-914]-K	6.25
685.97	599.94	E-[491-508]-R	7.06
693.84	979.55	E-[345-355]-K	5.10
748.42	229.12	E-[998-1010]-R	11.08
765.83	234.15	A-[241-254]-K	8.22
772.84	416.25	D-[771-784]-K	6.63
846.12	276.13	A-[724-745]-R	7.74
853.94	1034.55	A-[756-770]-K	9.13
940.48	277.12	E-[425-439]-K	6.90
1195.01	373.21	E-[162-181]-K	7.43
256.63	379.21	I-[1011-1018]-R	5.01
257.90	216.12	I-[154-161]-K	3.03
292.16	260.20	H-[210-217]-K	5.66
297.65	293.16	F-[105-113]-K	5.30
330.50	288.16	H-[855-862]-R	3.86
405.19	262.14	G-[182-188]-K	6.85
447.72	289.16	I-[885-891]-R	4.05
451.24	788.39	L-[622-628]-R	4.04
482.79	310.18	L-[941-948]-K	4.20
514.25	298,10	H-[509-520]-R	5.94
531.27	244.17	G-[313-322]-K	7.02
560.00	602.36	F-[395-408]-R	5.48
593.84	745.41	I-[1024-1033]-K	5.84
598.30	242.15	I-[746-755]-K	7.43
598.62	429.19	H-[1048-1061]-K	6.38
733.89	228.13	L-[830-841]-K	7.00
745.89	274.19	L-[81-93]-R	5.49
750.35	1058.51	F-[409-420]-K	8.79
756.38	618.32	I-[949-968]-R	10.00
763.90	243.13	I-[972-984]-K	6.62
764.07	869.48	L-[524-543]-R	5.53
442.27	468.28	S-[255-262]-R	4.68
475.89	659.35	M-[1-11]-K	5.61
477.56	254.16	R-[634-646]-K	4.35
478.27	277.16	L-[876-882]-K	4.38
498.25	569.24	V-[794-801]-K	2.89
500.75	218.15	M-[817-824]-K	5.56
514.75	798.38	M-[328-335]-K	4.45
522.29	831.42	V-[302-310]-K	4.34
583.31	669.85	V-[263-277]-K	5.52
604.95	394.18	S-[698-712]-K	5.65
657.82	304.16	S-[67-77]-R	8.38
723.83	347.19	Y-[379-391]-K	3.55
735.03	201.12	V-[189-208]-K	6.24
815.43	215.14	T-[802-816]-R	9.59
829.04	919.43	S-[282-301]-R	6,99
903.89	520.23	O-[559-588]-K	6.85
977.11	260.20	S-[114-139]-K	10.11
1151.55	375.20	N-[359-378]-K	7.31

B

1	MNHQQQQQQKAGEQQLSEPEDMEMEAGDTDDPPRITQNPVINGNVALSD	50
51	GHNTAEEDMEDDTSWRSEATFQFTVERFSRLSESVLSPPCFVRNLPWKIMVMP	103
104	RFYPDRPHQKSVGFFLQCNAESDSTSWSCHAQAVLKIINYRDDEKSFSRRISHLFF	159
160	HKENDWGFSNFMAWSEVTDPEKGFIDDDKVTFEVFVQADAPHGVAWDSKKH	210
211	TGYVGLKNQGATCYMNSLLQTLFFTNQLRKAVYMMPTEGDDSSKSVPLALQRV	263
264	FYELQHSDKPVGTKKLTKSFGWETLDSFMQHDVQELCRVLLDNVENKMKGTCV	316
317	EGTIPKLFRGKMVSYIQCKEVDYRSDRREDYYDIQLSIKGKKNIFESFVDYVAVEQL	373
374	DGDNKYDAGEHGLQEAEKGVKFLTLPPVLHLQLMRFMYDPQTDQNIKINDRFEF	427
428	PEQLPLDEFLQKTDPKDPANYILHAVLVHSGDNHGGHYVVYLNPKGDGKWCKFD	481
482	DDVVSRCTKEEAIEHNYGGHDDDLSVRHCTNAYMLVYIRESKLSEVLQAVTDHDI	536
537	PQQLVERLQEEKRIEAQKRKERQEAHLYMQVQIVAEDQFCGHQGNDMYDEEKV	589
590	KYTVFKVLKNSSLAEFVQSLSQTMGFPQDQIRLWPMQARSNGTKRPAMLDNEA	642
643	DGNKTMIELSDNENPWTIFLETVDPELAASGATLPKFDKDHDVMLFLKMYDPKT	696
697	RSLNYCGHIYTPISCKIRDLLPVMCDRAGFIQDTSLILYEEVKPNLTERIQDYDVSLDK	755
756	ALDELMDGDIIVFQKDDPENDNSELPTAKEYFRDLYHRVDVIFCDKTIPNDPGFVV	811
812	TLSNRMNYFQVAKTVAQRLNTDPMLLQFFKSQGYRDGPGNPLRHNYEGTLRDLL	865
866	QFFKPRQPKKLYYQQLKMKITDFENRRSFKCIWLNSQFREEEITLYPDKHGCVRDLL	922
923	EECKKAVELGEKASGKLRLLEIVSYKIIGVHQEDELLECLSPATSRTFRIEEIPLDQVDI	982
983	DKENEMLVTVAHFHKEVFGTFGIPFLLRIHQGEHFREVMKRIQSLLDIQEKEFEKFK	1039
1040	FAIVMMGRHQYINEDEYEVNLKDFEPQPGNMSHPRPWLGLDHFNKAPKRSRYTY	1093
1094	LEKAIKIHN	1102

Figure S4: USP-7 activity assay on GeGe-3 and P02277.
Evaluation of USP-7 inhibition by (A) GeGe-3, a close STIRUR 41 structural analog and by (B) the USP-7 known inhibitor P02277.

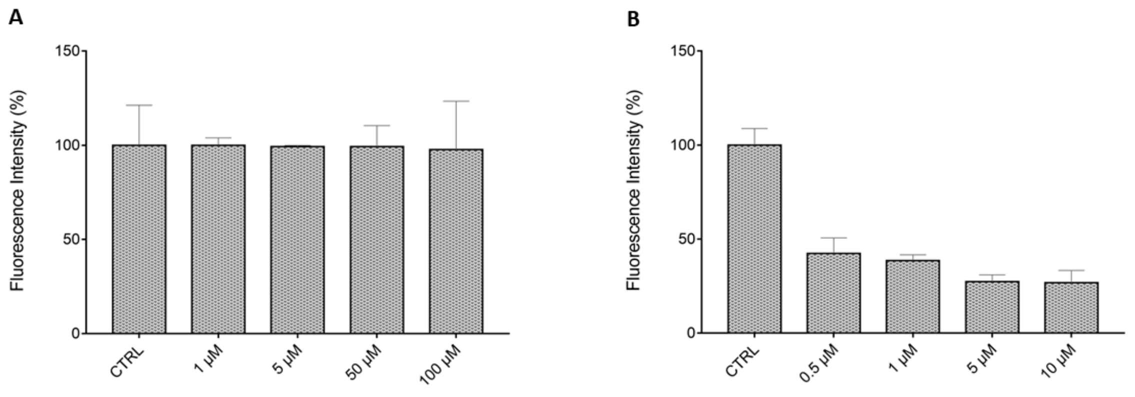

Figure S5: Evaluation of USP-7 expression in HTLA-230 cells treated with increasing amounts of P02277.
HTLA-230 cells were treated with increasing concentrations of P02277 and the lysates submitted to Western Blotting analysis. As observable, the molecule induces a decrement of USP-7 protein levels starting from 1.5 µM.

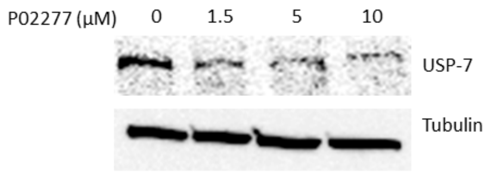

Figure S6: USP-7 C-terminal domain.
Structure of USP-7 C-terminal domain. The locations of the two putative binding sites are showed. Fragments identified by t-LiP-MRM are colored in green.

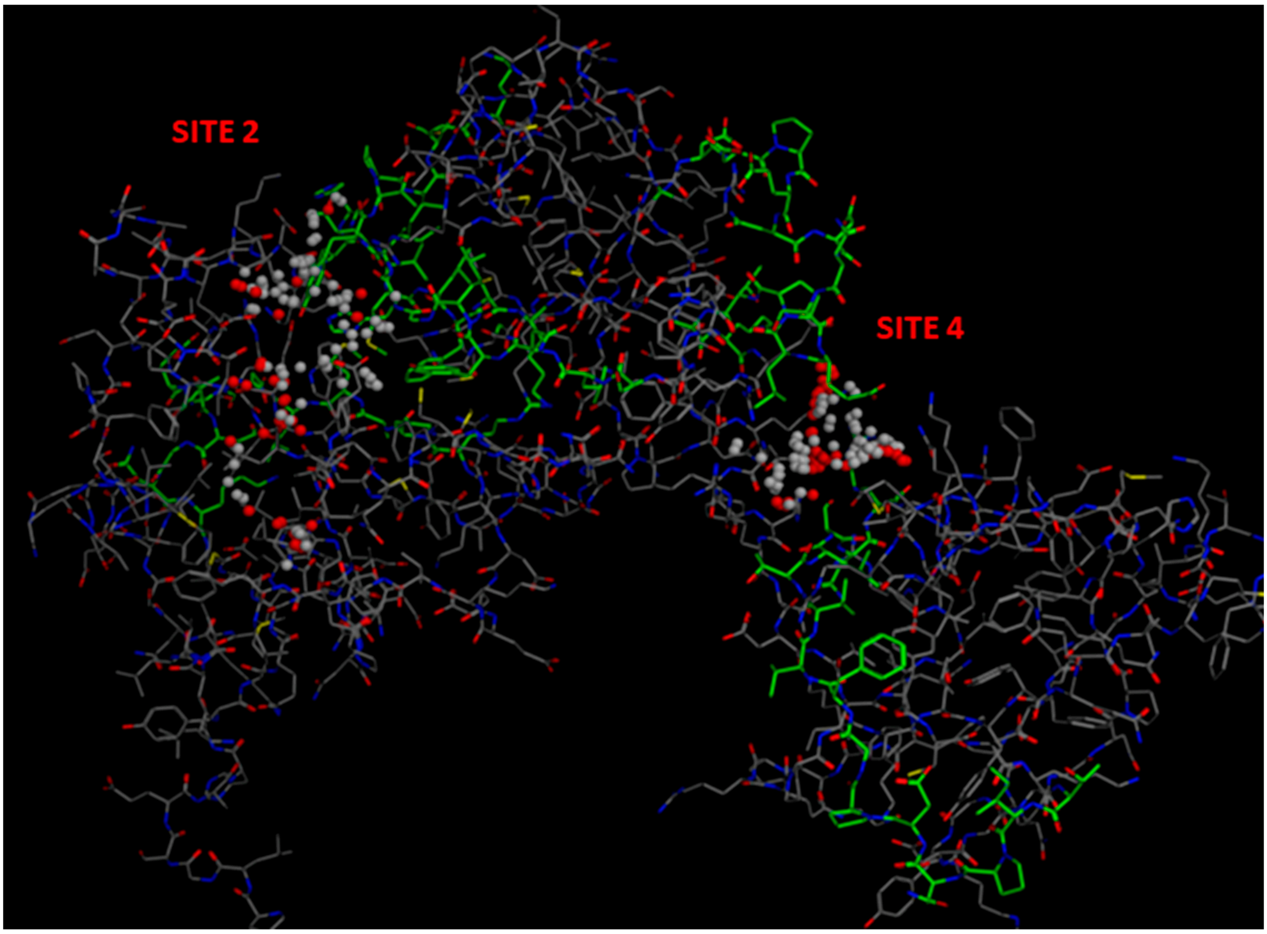

Table S1. **Size and residues lining the selected putative binding sites.**

	Size (Å²)	Residues
Catalitic site	110	TYR224, PHE291, MET292, GLN293, HIS294, ASP295, VAL296, GLN297, GLU298, GLN351, GLN405, LEU406, MET407, ARG408, PHE409, MET410, ASN418, LYS420, HIS456, ASP459, ASN460, HIS461, TYR465, TYR514
Site 2	124	ILE569, VAL570, ALA571, GLU572, ASP573, GLN574, CYS576, LYS590, TYR591, THR592, MET613, PHE615, PHE661, LEU662, GLU663, THR664, VAL665, ASP666, PRO667, LEU669, THR675, LEU676, PRO677, TYR701, CYS702, GLY703, HIS704, ILE705, TYR706, ARG723
Site 4	102	ARG697, GLU779, LEU780, PRO781, GLU785, ARG788, ASP789, HIS792, ARG793, THR812, LEU813, SER814, ARG816, MET817, GLN821, LYS824, THR825, GLN828
